# Modeling and Simulation of Robotic Grasping in Simulink Through Simscape Multibody

**DOI:** 10.3389/frobt.2022.873558

**Published:** 2022-05-31

**Authors:** Maria Pozzi, Gabriele Maria Achilli, Maria Cristina Valigi, Monica Malvezzi

**Affiliations:** ^1^ Department of Information Engineering and Mathematics, University of Siena, Siena, Italy; ^2^ Department of Engineering, University of Perugia, Perugia, Italy

**Keywords:** robotic grasping, robotic hands, multibody simulation, soft robotic hands, simulink, simscape, MATLAB

## Abstract

Grasping and dexterous manipulation remain fundamental challenges in robotics, above all when performed with multifingered robotic hands. Having simulation tools to design and test grasp and manipulation control strategies is paramount to get functional robotic manipulation systems. In this paper, we present a framework for modeling and simulating grasps in the Simulink environment, by connecting SynGrasp, a well established MATLAB toolbox for grasp simulation and analysis, and Simscape Multibody, a Simulink Library allowing the simulation of physical systems. The proposed approach can be used to simulate the grasp dynamics in Simscape, and then analyse the obtained grasps in SynGrasp. The devised functions and blocks can be easily customized to simulate different hands and objects.

## 1 Introduction

The numerical simulation of robot dynamics is widely diffused in research, industrial and education contexts. It can support the design of new devices from the very beginning, as well as the validation and comparison of different actuation and control systems. Simulations can also be employed to efficiently generate data for learning-based approaches. A comprehensive review of the available simulators for robotic systems has been recently presented in (Collins et al., 2021). As underlined by Tselegkaridis and Sapounidis (2021), simulators constitute also useful and accessible resources for teachers and educators. Eminent examples of software tools for simulating robots include the Robotics Toolbox available in MATLAB and Python (Corke, 2017; Corke and Haviland, 2021), Gazebo (Koenig and Howard, 2004; Qian et al., 2014), that is integrated in the ROS framework, and SOFA (Allard et al., 2007), designed for soft robots.

When it comes to the particular problem of simulating robotic grasping and manipulation, an effective software simulation environment should allow to model the used gripper, the grasped object, the environment, and their interactions. GraspIt! (Miller and Allen, 2004), OpenGRASP (Ulbrich et al., 2011), and SynGrasp (Malvezzi et al., 2015), are some of the few available simulators expressly designed for simulating grasping systems. All of them allow to define simulation environments including different hand and object models, but only SynGrasp has been explicitly designed for analysing underactuated and compliant grasps and is implemented in MATLAB. In robotic systems, the structural and mechanical behaviour is strictly related to actuation, sensing and control aspects, therefore a holistic approach is needed to properly design and analyse them. MATLAB has the advantage of being a widely used and powerful environment that allows managing all these aspects in an effective way, without requiring extremely advanced programming and modeling skills.

**FIGURE 1:**
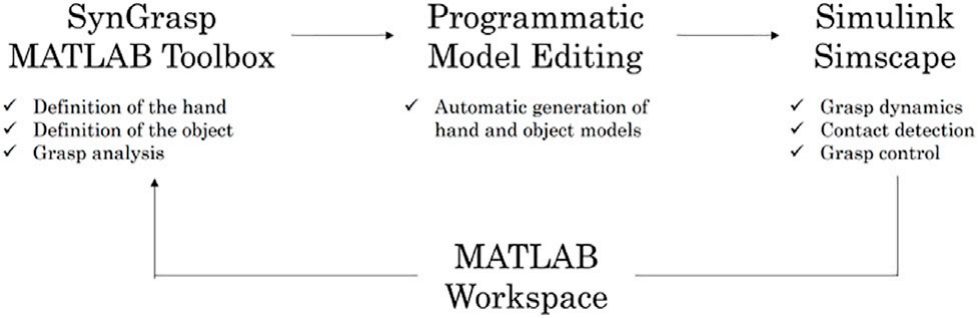
Fundamental components of the proposed approach.

This paper introduces a method to conduct the multibody simulation of grasping systems in a rather intuitive and user-friendly manner, exploiting widespread software tools like MATLAB™ and Simulink™. Building upon the SynGrasp Toolbox, in which hand and object models can easily be defined, we implemented a new toolbox of functions which connect SynGrasp to Simscape™ Multibody™ (MathWorks, Inc., Natick, Massachusetts, United States) through the programmatic creation of Simulink models. In this way, SynGrasp is enriched with the possibility of performing dynamic simulations, and the Simscape simulation outcomes can be seamlessly processed with the SynGrasp functions for grasp analysis. The proposed approach is illustrated in Figure 1.

The multibody simulation of mechanical systems is widely used in mechanical engineering for solving the mechanism’s kinematic and dynamic problems. Applications range from mechanical components (Logozzo and Valigi, 2019), to mechanisms (Valigi et al., 2020a) and robots (Achilli et al., 2021a; Zhu et al., 2022). The multibody model of a mechanical system is composed of rigid and deformable bodies, interconnected by means of kinematic pairs. Each body can undergo large translational and rotational displacements. The nonlinear analysis of mechanical systems can be performed by simulations considering dynamic loading conditions. Multibody simulators can also model the flexibility of bodies either by considering rigid bodies with lumped constraints, stiffness and damping to reproduce the deformability, or by means of models resulting from FEM (Finite Element Method) analyses (Shabana, 2003). These features can be particularly useful to simulate soft grippers and manipulators (Malvezzi et al., 2018, 2019; Pozzi et al., 2018; Achilli et al., 2020, 2021b; Valigi et al., 2020b).

Multibody simulation tools often offer the possibility of parametric modelling and the influence of the defined parameters on system dynamics can be investigated. This feature is particularly important in the first stages of product design, in which different solutions are investigated until the required performance is reached. In the successive phases of the development, parametric analysis is also important to study the effects of uncertainties and tolerances. Adams from MSC^1^ is one of the first and probably most famous commercial multibody dynamics simulation softwares, widely used in the study of the dynamics of mechanisms. Among open tools adopted in research contexts, Open Dynamics Engine^2^ is a very powerful and useful tool that allows creating, testing, and executing high-performance rigid body dynamics. OpenSim^3^, instead, is a free software that is widely used in research and rehabilitation centers for modeling and simulating human movement. MBDyn^4^ is another free simulation software for multibody dynamics, originally developed for aerospace applications. Another free multibody dynamics simulation software that is used both in research and industrial contexts is FreeDyn^5^. It is also worth to observe that multibody simulation is a specific package of more general CAE (Computer Aided Engineering) software, as for instance Ansys^6^ and Comsol^7^, or CAD systems like SolidWorks^8^.

In this paper, we adopted Simscape Multibody as it is fully integrated within the MATLAB framework and is thus easy to use in conjunction with the SynGrasp Toolbox. Simscape is also rather versatile, allowing the creation of models both in a programmatic way, and by manually assembling blocks. Notwithstanding Simscape Multibody is still less exploited than other simulation environments, also due to its more recent development, examples of robot modeling with Simscape have already been presented, including parallel (Olaya et al., 2017; Noskievič and Walica, 2020), industrial (Udai et al., 2011; Ahn, 2014; Le Ngoc and Nguyen, 2020), aerial (De Simone et al., 2017), legged (Eldirdiry and Zaier, 2018; Aldair et al., 2020), mobile (Khnissi et al., 2019; Siwek et al., 2019), and surgical (Taghizadegan et al., 2016) robots.

The paper is organized as follows. The main components and features of the proposed simulation framework are presented in Section 2. Results of grasp simulations conducted with the newly developed functions are reported in Section 3 and discussed in Section 4. Lastly, the current limitations and envisaged future developments of the toolbox are summarized in Section 5.

## 2 Materials and Methods

The basic elements of the presented framework are the SynGrasp toolbox (Malvezzi et al., 2015), Simscape Multibody, and the MATLAB Programmatic Model Editing functions. The latter allow to seamlessly connect the quasi-static model of a grasp defined in SynGrasp to its corresponding multibody dynamic model in Simscape. The presented framework is depicted in Figure 1 and described in the following, starting from its fundamental components.

### 2.1 Fundamental Components

#### 2.1.1 SynGrasp

SynGrasp is a MATLAB toolbox for grasp analysis of fully or underactuated robotic hands, in which lumped parameter models of compliant elements and synergistic coordination between joint variables can be modeled. Compliant and deformable elements can be modeled at contact points, in the joints or in the actuation system, including the transmission.

Hand structure and simulation conditions can be set by means of a Graphical User Interface or directly with a MATLAB script. The available functions allow to perform grasp evaluation in quasi-static conditions in terms of contact forces, actuator torques/forces, manipulability properties, grasp quality measures, and grasp stiffness. SynGrasp functions can be easily combined and modified to account for different grasping scenarios. Grasps can be described either using the provided grasp planner or directly defining contact points on the hand with the respective contact normal directions. Functions for the graphical representation of the hand and the object are provided. The toolbox is freely available at http://syngrasp.dii.unisi.it.

Since its release, SynGrasp has been used for analysing grasp properties of both human and robotic hands, for robotic hand design and also as a learning tool. In (Yao and Billard, 2020), the authors used the anthropomorphic hand model provided in SynGrasp to develop a kinematic procedure for estimating hand postures in bimanual tasks, whereas in (Fattahi Sani et al., 2019), the toolbox was used to develop a hand tracking system in surgical operations. Katyara et al. (2021) investigated the role of postural synergies in manipulation tasks and used SynGrasp to validate the proposed framework. Garate et al. (2017, 2018) exploited SynGrasp to implement in-hand manipulation strategies with a fully actuated hand based on Common-Mode Stiffness (CMS) and Configuration-Dependent Stiffness (CDS) principles, and to simulate control strategies for hand-arm systems subject to underactuation constraints (Ruiz Garate and Ajoudani, 2020). Using SynGrasp, Almeida and Moreno (2020) further developed the concept of Potential Grasp Robustness (Pozzi et al., 2017), devising new heuristics for managing computational load, and analysing the influence of uncertainties on its computation (Almeida and Moreno, 2021). Hybrid Cartesian-joint mapping procedures for anthropomorphic hands were analysed with SynGrasp by Meattini et al. (2021). Orabona et al. (2020) exploited the possibility of modeling underactuated hands in SynGrasp, to design a 3D-printed hand with a simple, yet versatile, structure. Zaidi et al. (2017) presented a method to model robotic hands grasping 3D deformable object, and SynGrasp functions were used for the graphical representation of grasps.

#### 2.1.2 Simscape

As previously introduced, multibody simulation aims at solving the kinematic and dynamic problems of mechanical systems, and is particularly useful to study the behaviour of complex mechanical and robotic systems like grippers and manipulators (Achilli et al., 2021a).

The main parameters used to describe bodies in the multibody simulator are: a local reference frame having the origin in the body’s center of mass and the principal axes, the mass, the tensor of inertia in the local reference frame, and other auxiliary references to define the constraints that are represented by relationships between the motion parameters. Both rigid and flexible bodies are modeled with a single or combining multiple blocks able to describe their mechanical behaviour. All bodies are connected by means of joints or with proper constrains and all is assembled into an articulated mechanism, with a number of degrees of freedom resulting from the kinematic relationships provided by the constraint blocks.

Several tools for performing multibody simulations are available. In this paper, we chose to exploit the application Simscape Multibody, as it is a MATLAB toolbox and can thus be easily integrated with SynGrasp. Simscape Multibody can use data from external 3D CAD, like SolidWorks (Dassault Systèmes, Vélizy-Villacoublay, France), to import 3D models obtained from 3D scanning (Valigi et al., 2020b), and to create bodies, constraints, actions, and joints with parametrization by mathematical expressions described in MATLAB. The bodies are characterised by their geometry and consequent inertial properties, which can be modified by the user according to the studied physical system. Forces and moments can be applied to bodies, and contact constraints can be defined. Simscape allows to model physical systems by connecting blocks representing different mechanical elements. Simscape and Simulink blocks can be mixed inside a model and linked through appropriate connectors. In the multibody system presented in this work, hand models are composed of subsystems including more than one block, and contact constraints are defined between the object to be grasped and the gripper’s phalanges and palm.

#### 2.1.3 Programmatic Model Editing

The programmatic modelling functions available in MATLAB allow to create and edit Simulink models programmatically, i.e., through a MATLAB script^9^. This feature is particularly suited for our objective of connecting SynGrasp, which is natively composed of MATLAB scripts and functions, with Simulink models including Simscape Multibody blocks. Through rather intuitive functions it is possible to programmatically add and connect blocks in models and set their parameters and properties.

In this work, we defined a library of basic Simscape Multibody subsystems (i.e., sets of blocks) that can be combined to create hand and object models (see Section 2.2). Based on the system that must be simulated, these blocks can be added to a new model and their parameters can be set according to the envisaged application.

### 2.2 Description of the Toolbox

The proposed toolbox is composed of a library of basic blocks in.slx format and a set of MATLAB functions aimed at manipulating them to create custom grasping systems, i.e. systems with a custom hand and a custom object that can interact with each other. In this work, we focus on grasping tasks, but the toolbox could be expanded to simulate also in-hand manipulation strategies (Prattichizzo et al., 2020).

Table 1 briefly describes the functions and the blocks of the new toolbox, which are available together with demonstrative scripts at http://syngrasp.dii.unisi.it. In the following, we first describe how the two main entities of a grasp, i.e., the hand and the object, are defined in the proposed framework, and then we detail how a grasp can be simulated.

**TABLE 1:** Implemented MATLAB functions and Simulink blocks.

Name	Description
SG2Sim_Hand.m	Defines the Simscape model of a hand
SG2Sim_Palm.m	Defines the Simscape model of the palm of a given hand
SG2Sim_Finger.m	Defines the Simscape models of the fingers of a given hand
SG2Sim_BuildObject.m	Defines the Simscape model of an object
SG2Sim_Contacts.m	Given a hand and an object defines contact contraints between them
SG2Sim_SimulateGrasp.m	Simulates the closure of the hand over an object and stores the
	achieved contact points and joint angles in the SynGrasp hand structure
SG2Sim_StartConfiguration.slx	Contains the World frame, the Solver Configuration and the
	Mechanism Configuration
SG2Sim_module_RJ.slx	Contains the module representing the finger phalanx
SG2Sim_PalmBlock.slx	Contains palm blocks configured to support a different number
	of fingers
SG2Sim_NoPalm.slx	This block is loaded in case the fingers have coincident bases
SG2Sim_Object.slx	Models different objects
SG2Sim_ExportData.slx	Allows to export simulation outcomes to the Workspace

#### 2.2.1 Hand Definition

**FIGURE 2:**
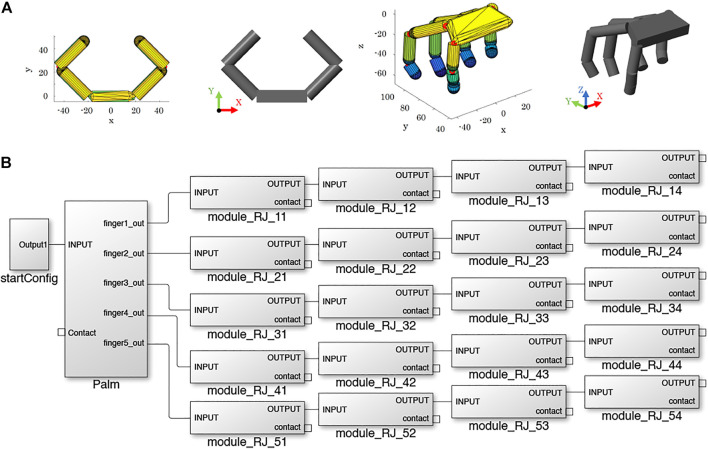
Hand definition. **(A)** Examples of hand models in SynGrasp (coloured) and in Simscape Multibody (grey): planar gripper with 2 fingers and anthropomorphic hand. **(B)** Simulink model of the anthropomorphic hand.

In SynGrasp, hand models are defined based on the Denavit-Hartenberg (DH) parameters of the fingers (Siciliano et al., 2009), which are considered to be open kinematic chains with several links connected through revolute joints (Malvezzi et al., 2015)^10^. The fingers are connected to a palm that is built starting from the positions of the fingers’ bases. In the MATLAB workspace, SynGrasp hands are represented as structures with several fields (e.g., number of fingers, number of joints, etc.). The function *SG2Sim_Hand* takes as input a hand structure and builds the corresponding hand model in Simscape Multibody, starting from its palm (*SG2Sim_Palm*) and then defining the fingers (*SG2Sim_Finger*). One of the simplest and one of the most complex hand models available in SynGrasp and their Simscape Multibody counterparts are shown in Figure 2A.

**FIGURE 3:**
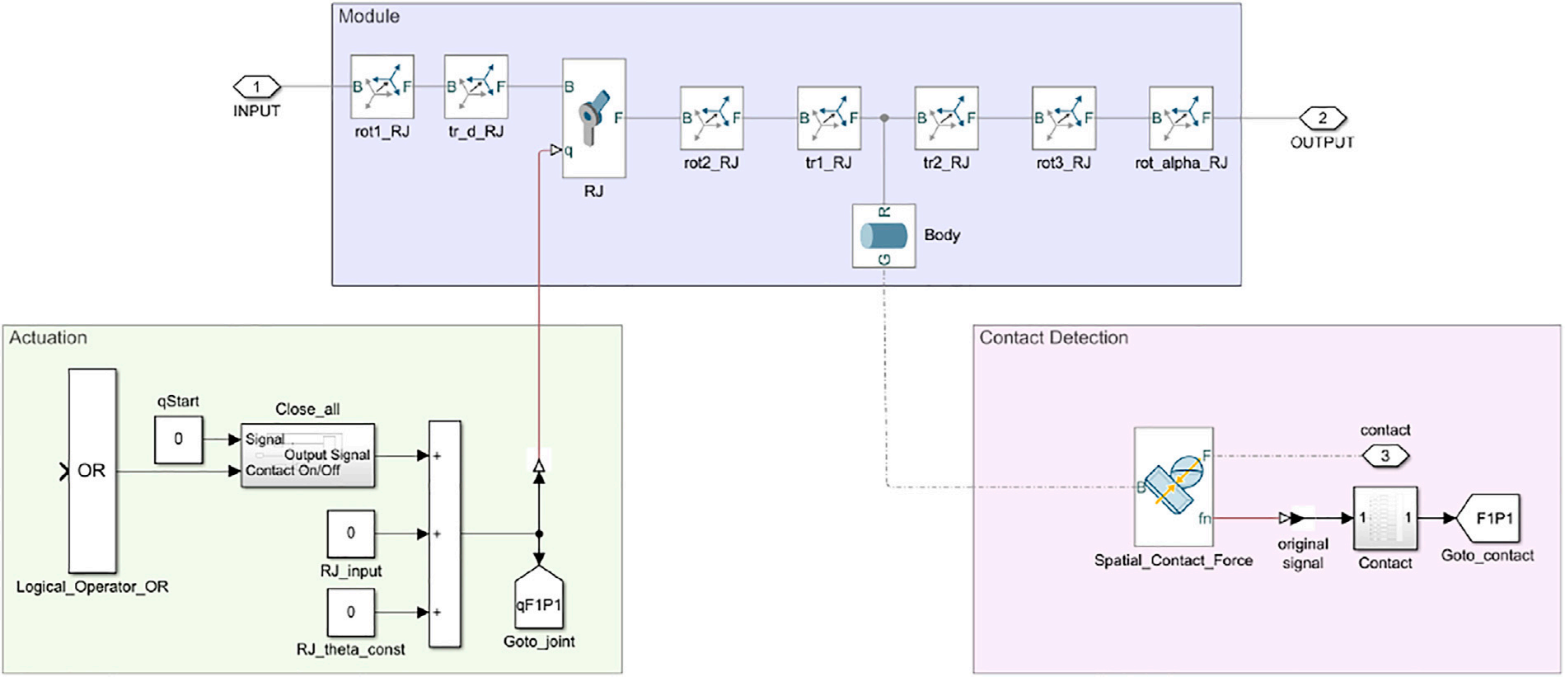
Phalanx module. Each phalanx is composed of a revolute joint and a rigid link connected through suitable transformations (purple). This figure also shows the phalanx actuation system (green) and the blocks for the contact detection (pink).

The creation of a hand in Simscape Multibody follows a modular approach. The hand is made of a palm and one or more fingers, composed of one or more phalanges. An example of hand structure is shown in Figure 2B. Depending on the number of fingers, a certain palm structure is loaded in the Simulink model through the function *SG2Sim_Palm*, and then phalanx modules are assembled to model the fingers with *SGSim_Finger*. Each phalanx has the same structure and is called *module_RJ_xy*, where *x* indicates the finger and *y* indicates the phalanx. As shown in Figure 3, a phalanx module contains a *Body* representing the phalanx^11^, a revolute joint representing the joint at the base of the phalanx, and several rigid body transformations blocks, which implement the DH rotations and translations. In hand models having two joints placed at the same position, the *SGSim_Finger* function automatically comments out the *Body* block of the first involved phalanx. In this way, the approach can flexibly handle different hand models, while keeping a modular structure. The anthropomorphic hand shown in Figure 2, for example, has two revolute joints at the base of each finger to account for flexion/extension and adduction/abduction motions (Gioioso et al., 2013; Marullo et al., 2022). Thus, in the first phalanx of each finger in the Simscape model, the *Body* block is commented.

Each phalanx module also contains the subsystems implementing the contact detection and the actuation system (see Section 2.2.3.1 and Section 2.2.3.2 for more details).

#### 2.2.2 Object Definition

**FIGURE 4:**
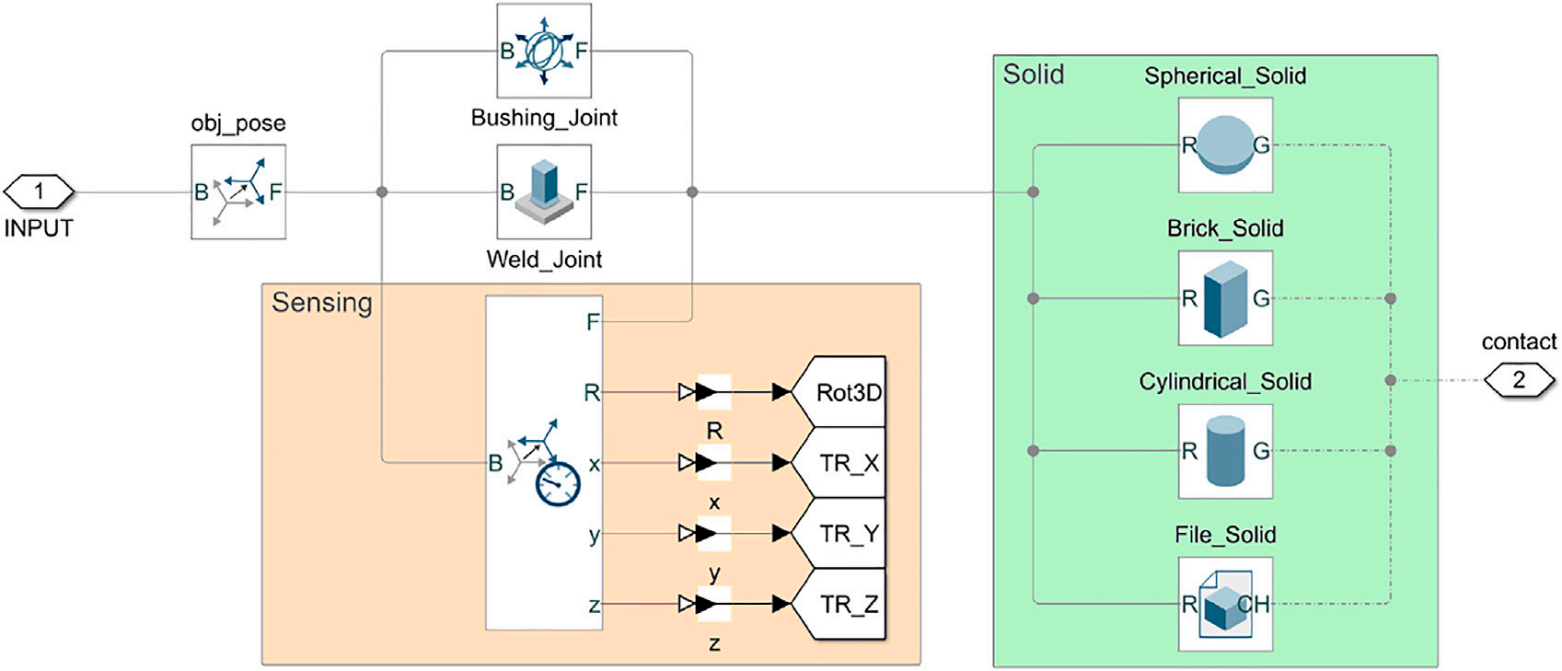
Object module. Each object can either be fixed in the workspace or free to move and is represented by a solid (green). The latter can either have a primitive shape (sphere, cuboid, cylinder) or be loaded from an external object model. The object module also contains the blocks devoted to sensing its pose (orange).

In the proposed framework, the object to be grasped is first defined in SynGrasp, where the main parameters are set, and then inserted into Simscape thanks to the function *SG2Sim_BuildObject*. The *Object* subsystem (Figure 4) contains: *1*) a rigid body transformation that sets the pose of the object, *2*) a joint that chooses the allowed object motions (e.g., a *Weld Joint* fixes the object in the workspace, whereas a *Bushing Joint* allows its unconstrained motion in 3D), *3*) a subsystem for sensing the object pose, and *4*) a solid block where the geometrical and material properties of the object can be specified.

The object to be inserted in the model can be chosen from a set of four different types of solid bodies: three primitive solids, *Cylinder*, *Parallelepiped* or *Sphere*, and a 3D solid, whose model can be imported by the user. For primitive solids, the main parameters (e.g., radius, height, length, etc.) must be specified either manually or exploiting the function *SG2Sim_BuildObject*, which sets them based on the object structure defined in SynGrasp. The *File_Solid* block, instead, allows the user to load a file in common formats used in 3D modeling such as STL, SAT, JT and STEP, but also proprietary software formats such as CATIA, Creo, Inventor, Unigraphics NX, Solid Edge, SolidWorks, and Parasolid. All four types of solid body blocks require *Inertia* parameters to be entered, where the mass or density of the object can be indicated.

#### 2.2.3 Grasp Simulation

Once a hand and an object are defined in the Simulink model file, contact constraints between them need to be defined through the function *SG2Sim_Contacts*. Then, a grasp can be simulated by activating the closure of the hand through *SG2Sim_SimulateGrasp*.

##### 2.2.3.1 Contact Definition and Detection

The interaction between solids within the multibody simulator is an important aspect for the grasping evaluation. The Simscape Multibody toolbox contains a block dedicated to this function, called *Spatial_Contact_Force*. This block allows to develop a contact model between a pair of bodies. Among the contact-force models in multibody dynamics (Skrinjar et al., 2018), Simscape Multibody uses as default the Kelvin-Voigt model, which was used in (Machado et al., 2012; Corral et al., 2021). The model is based on a regularized approach, also known as compliance or visco-elastic method, in which the contacting bodies are considered to be deformable at the contact zone, and the contact forces can be expressed as a continuous function of the local deformation between the contacting surfaces. In this block, the normal contact force F_N_ is estimated through the interpenetration between the solid bodies:

$$F_{N}=\;K\delta \;+\;D\dot{\delta }$$

where *K* is the stiffness coefficient and *D* is the damping coefficient, *δ* is the relative indentation between the contacting bodies, and $\dot{\delta }$ is the relative normal contact velocity. The first term is referred to the linear elastic force component, while the second one accounts for the energy dissipation during contact (Shigley, 1972). The normal contact force is proportional both to the indentation velocity and depth.

Another parameter that can be set in the *Spatial_Contact_Force* block is the transition region width, which characterizes the transitional region to the force equations ^12^. As the depth of indentation gets to the end of the transition region, the normal force increases, until the maximum stiffness and damping forces are reached. By varying the transition region width, the transition is sharper for low values, while it is smoother for high values.

The main parameters of the contact model and the corresponding values adopted in the developed Simscape subsystems are shown in Table 2.

**TABLE 2:** Contact model: main parameters.

Normal Force	
Stiffness	10^6^ *N*/*m*
Damping	10^3^ *N*/(*m*/*s*)
Transition region width	10^−6^ *m*
**Frictional Force**	
Method	Smooth Stick-slip
Coefficient of static friction	0.5
Coefficient of dynamic friction	0.3
Critical velocity	10^−3^ * m*/*s*

The *Spatial_Contact_Force* block uses a sensing output system, which shows how certain calculated parameters vary between the two solids in contact. The sensing output parameters available in this module allow to monitor the separation distance between the two solid elements, the modulus of the normal contact force between the two elements of the solid body, and the modulus of the frictional contact force between the two elements of the solid body.

The function *SG2Sim_Contacts* allows to connect each phalanx of the hand model and the palm to the object via a *Spatial_Contact_Force* block (see Figure 3). The modulus of the normal contact force is selected as the parameter to establish the presence of contact between bodies. A threshold can be chosen beyond which it can be considered that the interaction between the solid elements is present, and thus a contact is detected. The contact module contains a logic that evaluates the ten previous samples of the measured normal contact force and compares them: if all the samples are higher than the selected threshold, the contact can be considered as established. The signal, passing through ten *Delay* blocks, is processed in a *Product* block: if a sample is lower than the threshold, the output value is 0, otherwise it is 1. The number of *Delay* blocks used in the subsystem is obtained from experimental trials during the development of the toolbox. This control system is necessary to avoid that incorrect values of the modulus of the normal force are considered as established contacts. In fact, there are cases in which peaks in the normal force values occur due to the contact model main parameters. Indeed, the combination of stiffness and damping limits the possibility of interpenetration between the bodies; thus, the presence of rebounds between the surfaces is possible, but it cannot be considered as an established contact between solid bodies.

Each Contact subsystem output port ends in a tag block. These tag blocks are handles: they send the data present at the head of the *GoTo* block (sender block) to that present in the *From* block (receiver block). Each tag block is named as *FxPy*, where *x* indicates the finger and *y* indicates the phalanx, in accordance with the naming of phalanx modules. Notice that two types of signals are used in Simscape Multibody: the Physical type and the Simulink type. Only Simulink signals can be sent with tags, and this is why *Simulink-PS Converter* blocks are used to connect Simulink sources to inputs to a physical Simscape Multibody network (see Figure 3).

Tags are used throughout the model to send data in a user-friendly and intuitive way, avoiding the large number of lines that would otherwise be needed to achieve the same result without them. The data obtained after a simulation are stored in the *SG2Sim_ExportData* subsystem and sent to the MATLAB Workspace.

##### 2.2.3.2 Hand Closure

As hand models are composed of links connected through revolute joints, the actuation systems that can be implemented are either based on angular position inputs or based on torque commands. In this work, we adopted the first option. Once a hand and an object are defined in the Simulink model file, and so are the contacts between them, a grasp can be simulated by activating the closure of the hand. To this aim we created an ad-hoc subsystem implementing the position control of the hand fingers which is activated by the function *SG2Sim_SimulateGrasp*. Starting from a predefined initial configuration, the hand actuation system closes all the joints of the hand over the object, increasing them by a pre-defined constant value which can be specified for each joint.

The increment in the joint value is applied only if no contact is detected on the related phalanx or on the successive ones. To implement this logic we adopted a *Logical_Operator_OR* that is present in each phalanx module (see Figure 3). In case a contact is detected, the phalanx remains in the same position. With a delay block, the joint angle of the previous instant is stored throughout the simulation. In this way, if the object and the phalanx are no longer in contact due to the object motion, the actuation system restarts increasing the joint position until the next contact occurs.

## 3 Results

The presented framework was tested in two sets of simulations: *1*) grasps of objects with different shapes, and *2*) grasps of objects made of different materials.

In all the simulated grasps, the hand was closed over the object using the actuation system detailed in Section 2.2.3.2, and contacts were detected with the strategy described in Section 2.2.3.1. Objects with primitive shapes (sphere, cylinder, cuboid), as well as object models from the YCB Dataset (Calli et al., 2015) were considered. The effect of gravity was deactivated, to show the working principles of the proposed contact detection and actuation strategies without external loads.

For the simulations, an explicit continuous fixed-step solver was chosen. Different solvers of this type are available in Simulink, according to the used numerical integration technique. As the order of the integration technique increases, also the computational complexity grows. We adopted the Ode3 solver, which computes the model state using the Bogacki-Shampine formula integration technique to evaluate state derivatives (Hosea and Shampine, 1994). Among the integration algorithms available in Simulink, we selected a fixed-step one also to simplify the post-processing phase and the analysis of the results (e.g. analysis in the frequency domain). The simulations described in this section were conducted with MATLAB R2021b version on a Laptop with Windows 10, Intel^R^ Core™ i7-10750H CPU @2.60GHz, and 16 GB RAM.

To increase the accuracy of the simulation, the model requires to use a step size that is as low as possible. The balance between solver order and step size can only be obtained through iterative trials, to achieve accurate results with a low computational burden. Furthermore, to simulate the grasp with generic objects, the contact model requires a high number of steps to avoid conditions in which the solution diverges. Through numerous tests, the value 10^−5^ *s* was identified as a suitable step size, that gives accurate solutions in a reasonable amount of time, and with several different objects.

**FIGURE 5:**
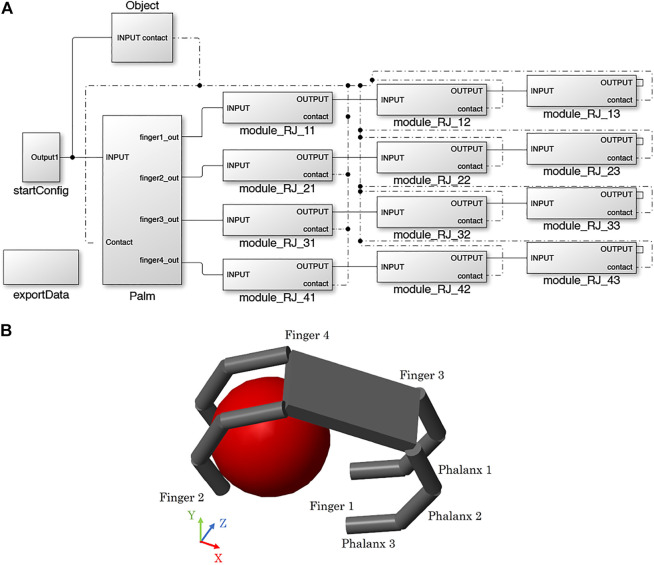
Four fingered gripper grasping a sphere: block scheme **(A)** and structure **(B)**.

In the next sections the results obtained with a four fingered gripper having the structure shown in Figure 5 are described. However, the conducted simulations can be easily performed with other types of hands.

### 3.1 Objects With Different Shapes

**FIGURE 6:**
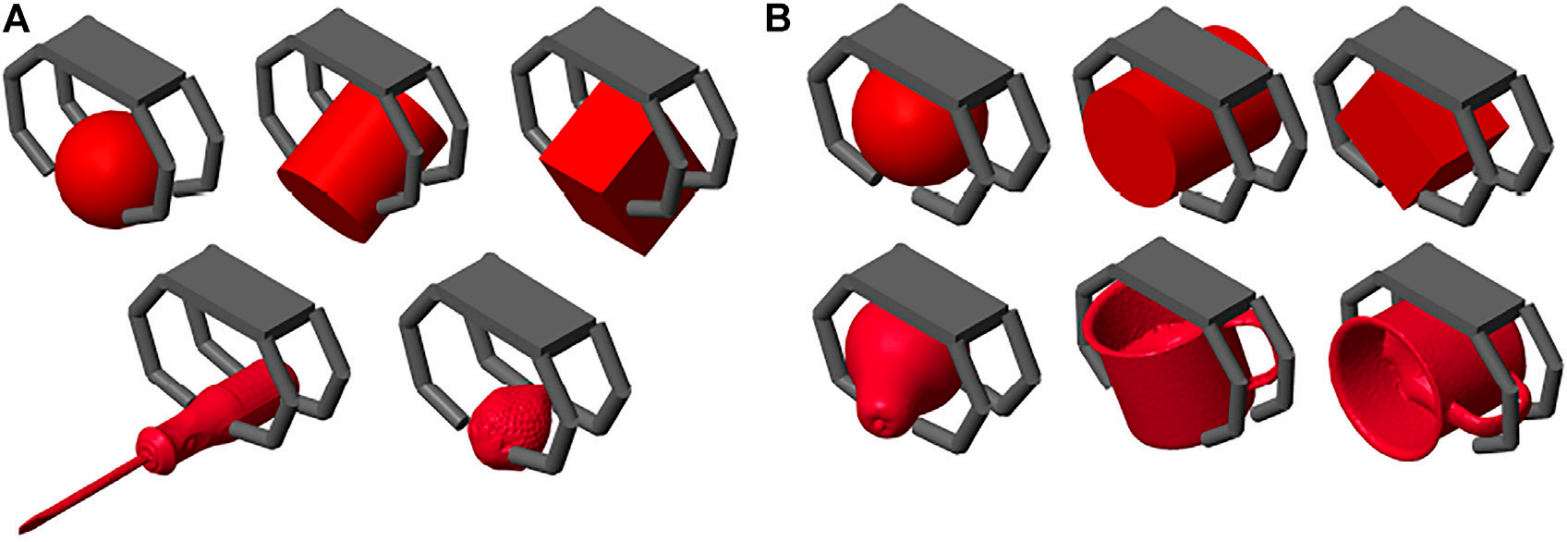
Performed simulations. **(A)** Precision grasps. **(B)** Power grasps.

To simulate precision grasps we fixed all the object degrees of freedom through a Simscape Multibody Weld Joint, so that even when touched by the hand the object did not move, allowing the precise positioning of the fingertips over it. Examples of the obtained final grasps are shown in Figure 6A.

To simulate power grasps, instead, the object was left free to move in the 3D space. To this aim, the Simscape *Object* block was attached to the *startConfig* block through a Bushing Joint that has three translational and three rotational degrees of freedom. As a result, while closing over the object, the hand could drag it towards the palm, ending up in a power grasp involving the phalanges of the fingers as well as the palm itself. Examples of achieved power grasps are depicted in Figure 6B.

Both types of grasps were performed over primitive shapes (sphere, cylinder, cuboid). In addition, for precision grasps we chose two small objects from the YCB Dataset, i.e., the screwdriver and the strawberry, whereas for power grasp we chose two larger objects, i.e., the metal mug and the pear.

**FIGURE 7:**
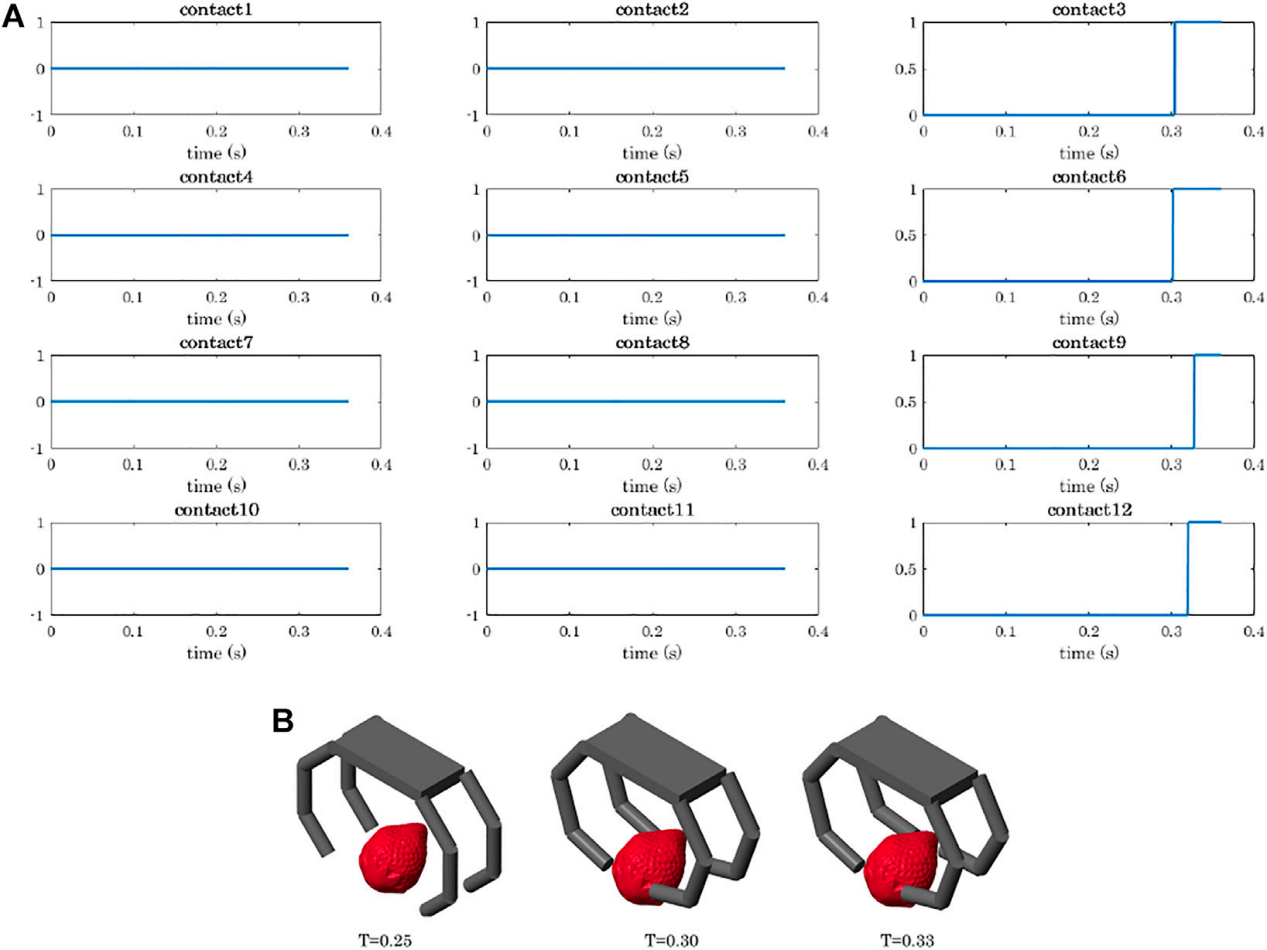
Contact detection during the precision grasp of a strawberry. **(A)** Contact signals ordered as the phalanges of the hand: the rows correspond to the fingers (from 1 to 4), and the columns to the phalanges (from 1 to 3). When the signal is equal to 0, it means that no contact has been detected on the corresponding phalanx. When a contact is detected the signal goes to 1. **(B)** Grasp sequence: Fingers 1 and 2 enter in contact with the object before Fingers 3 and 4.

In precision grasps, objects were oriented in different ways and positioned so that they would enter in contact first with the hand fingertips, thus achieving a pinch grasp. Figure 7 shows the results obtained for the grasp of the strawberry in terms of contact detection and performed motion.

**FIGURE 8:**
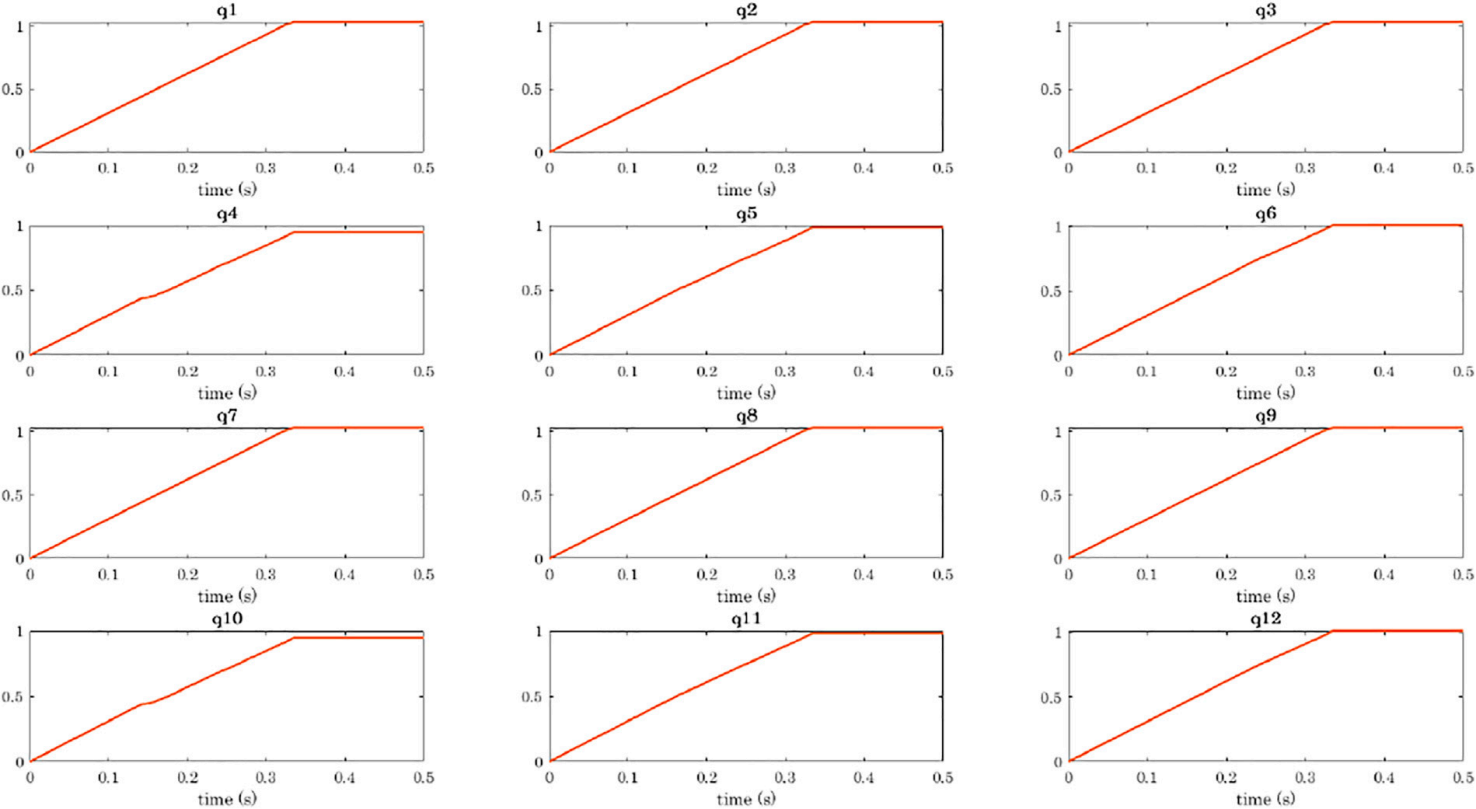
Power grasp of a sphere with the four-fingered gripper: trajectories of the joints. Plots are ordered as the phalanges of the hand: the rows correspond to the fingers (from 1 to 4), and the columns to the phalanges (from 1 to 3).

**FIGURE 9:**
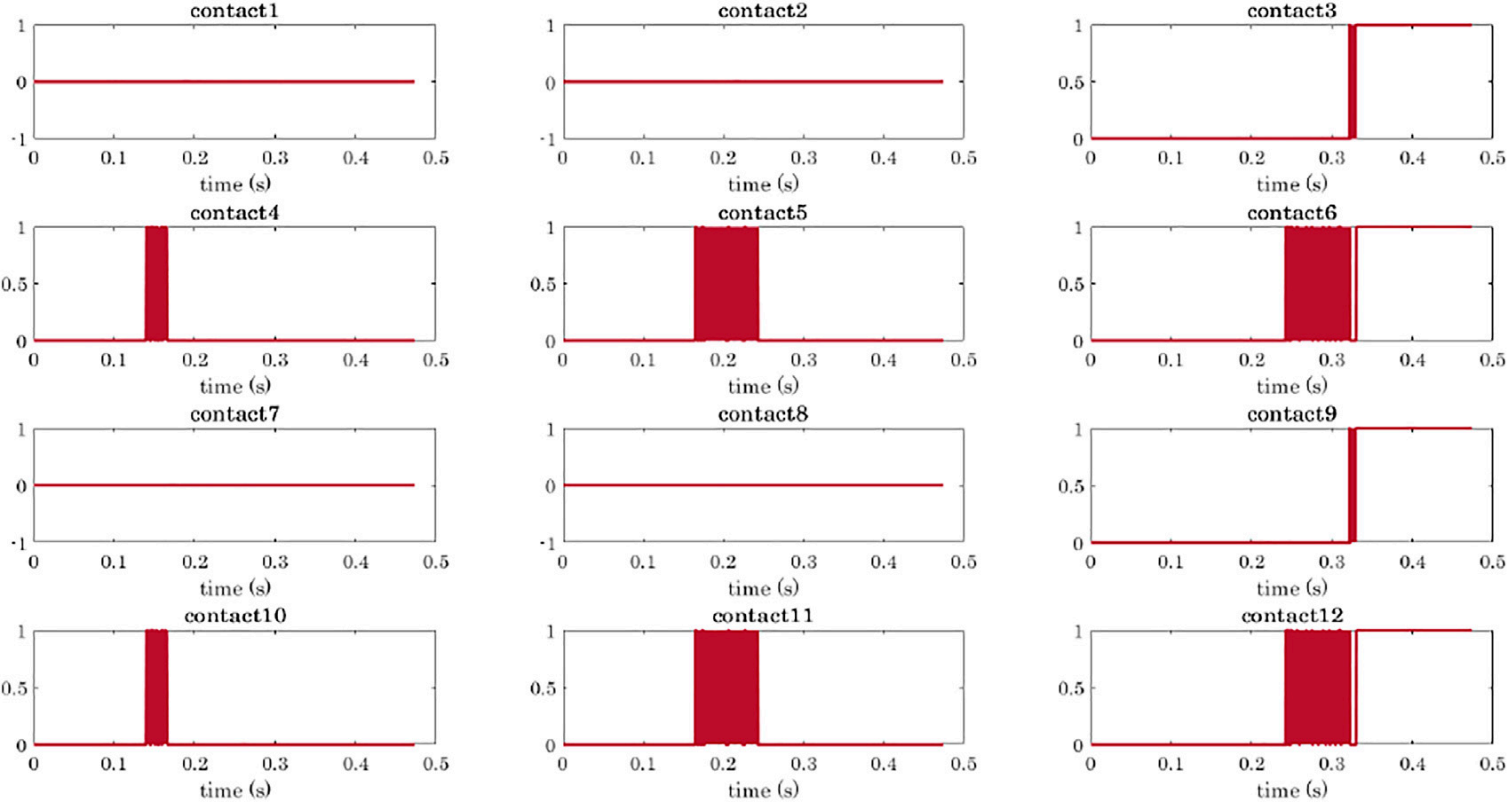
Power grasp of a sphere with the four-fingered gripper: contact detection. Contact signals are ordered as the phalanges of the hand: the rows correspond to the fingers (from 1 to 4), and the columns to the phalanges (from 1 to 3). When the signal is equal to 0, it means that no contact has been detected on the corresponding phalanx. When a contact is detected the signal goes to 1.

**FIGURE 10:**
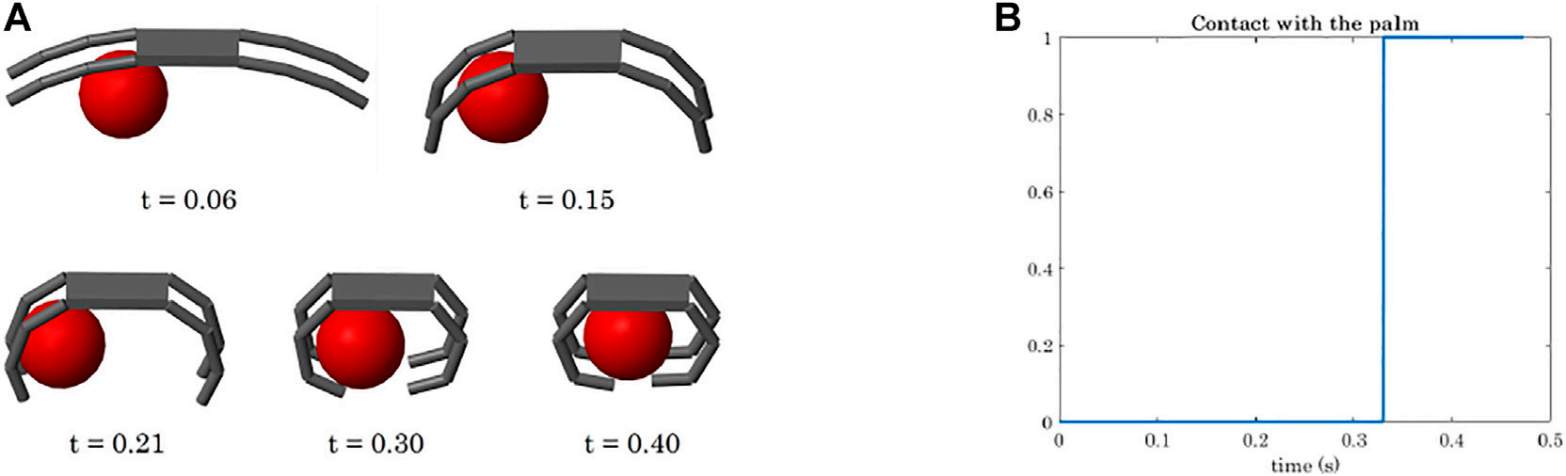
Power grasp of a sphere with the four-fingered gripper: temporal sequence of the closing motion **(A)**, contact detection on the palm **(B)**.

**FIGURE 11:**
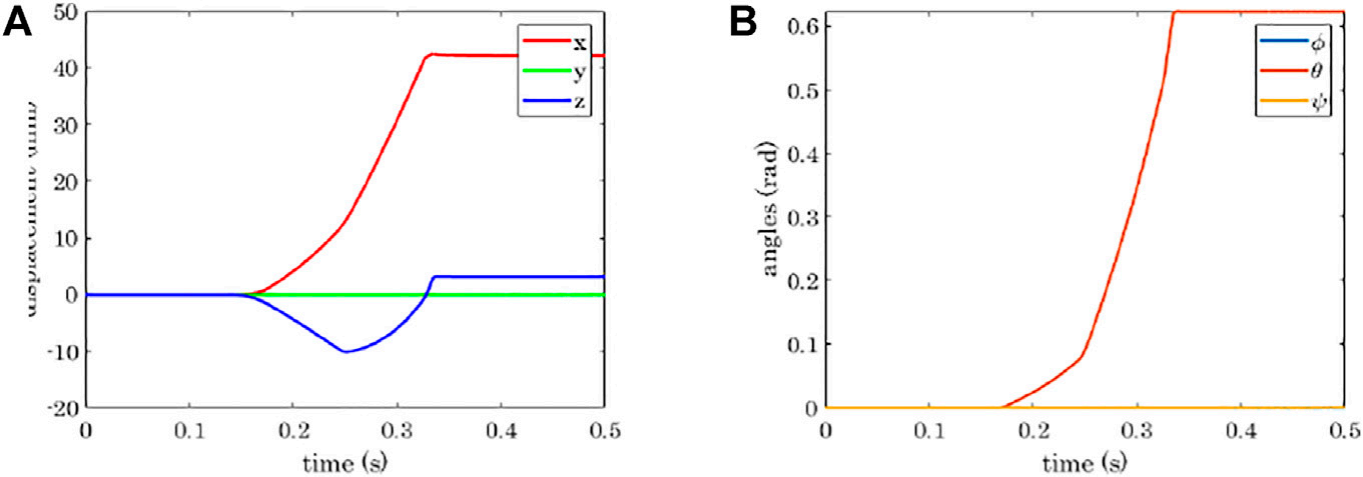
Power grasp of a sphere with the four-fingered gripper: position coordinates indicating the object translation **(A)**, and Euler angles indicating the object rotation **(B)**. *ϕ*, *θ*, *ψ* indicate the rotation around *z*, *y*, and *x*, respectively.

The simulation of a power grasp of a sphere is analysed in terms of joints trajectories, contact detection, and object motion in Figures 8–11.

### 3.2 Objects Made of Different Materials

**FIGURE 12:**
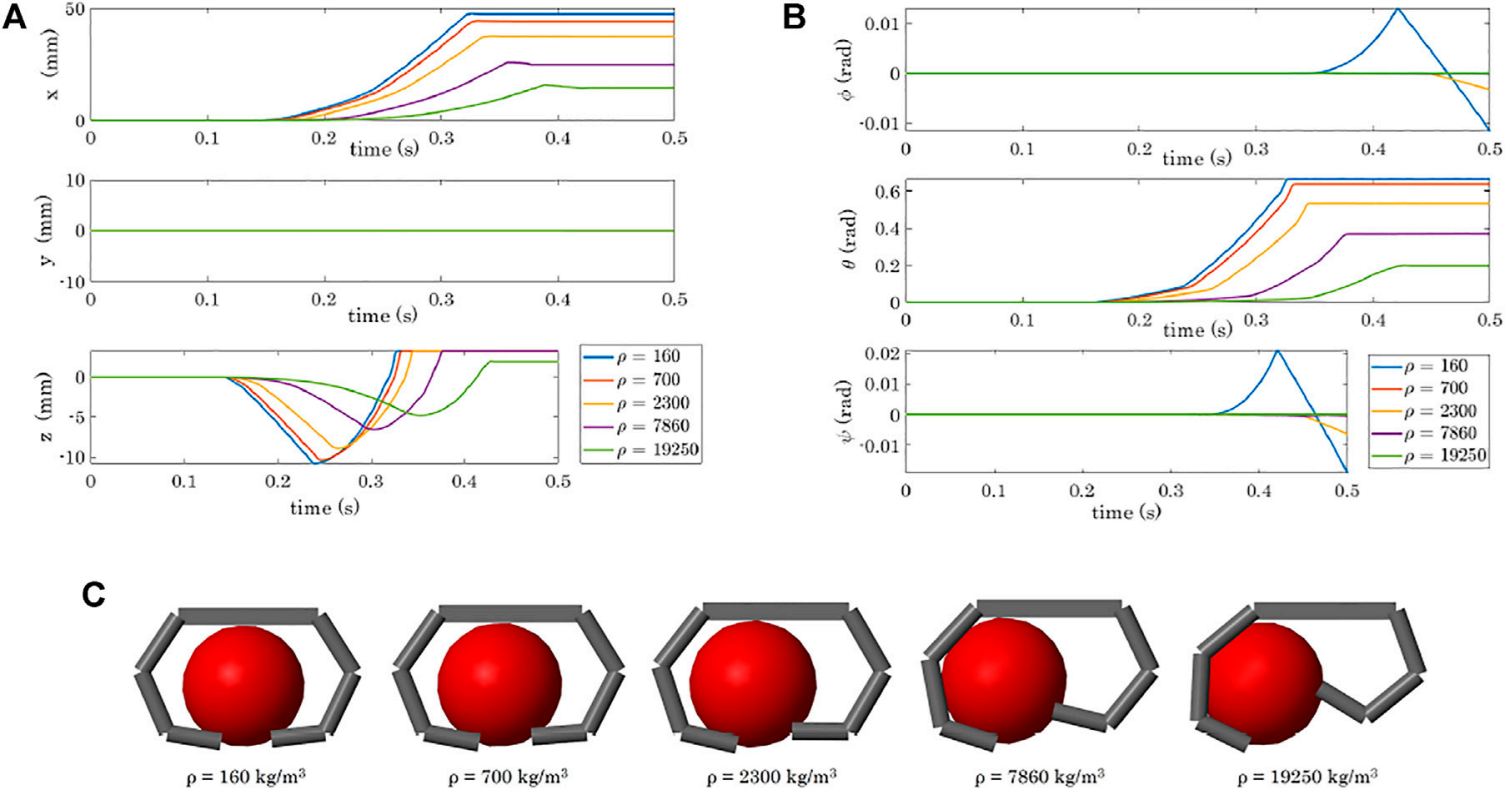
Variation of the object pose depending on the object density: position **(A)** and orientation **(B)**. **(C)** Obtained final grasps in the 5 simulations.

To fully show the capabilities of the multibody simulator, we tested the grasp of objects with different realistic densities: *ρ* = 160 kg/*m* ^3^ (balsa wood), *ρ* = 700 kg/*m* ^3^ (fir wood), *ρ* = 2,300 kg/*m* ^3^ (chalk), *ρ* = 7,860 kg/*m* ^3^ (steel), *ρ* = 19250 kg/*m* ^3^ (gold)^13^. We used the same gripper and spherical object adopted in the previous simulations and we initially placed the sphere as in Figure 10A. Figure 12 shows the results obtained varying the density of the spherical body in terms of object position and orientation and of final grasps.

## 4 Discussion

### 4.1 Objects With Different Shapes

As shown in Figure 6, the developed tools allow to simulate the grasp of objects with different shapes. We decided to present the results obtained for the strawberry, as they clearly show the working principle of the proposed grasping strategy. As soon as a finger touches the object with its fingertip, and the contact is stable, the whole finger stops moving. As depicted in 7(B), the Fingers 1 and 2 come into contact with the object slightly before the others. As a result, the contact signals corresponding to the last phalanges of Fingers 1 and 2 activate (passing from 0 to 1) slightly before other ones.

To show the capability of the simulator to compute the object motion when the object is free to move (even though not subjected to gravity), we simulated power grasps in which the object is dragged by the fingers towards the hand palm. We reported the data related to the grasp of the sphere, as already with a simple object we can appreciate different aspects of the simulation. The sphere was initially positioned closer to Fingers 2 and 4 as shown in Figure 5B. As a result, the first contacts are detected on the first phalanges of these two fingers (contacts 4 and 10, Figure 9). While closing, Fingers 2 and 4 go in contact also with their second phalanges (contacts 5 and 11) and lastly with the fingertips (contacts 6 and 12). At *t* ≈ 0.32 s, the contact with the last phalanges of Fingers 1 and 3 (contacts 3 and 9) is detected, and from *t* ≈ 0.33 s it becomes stable. Figure 10A shows the main steps of the grasping task. From *t* ≈ 0.33 s the contacts on the fingertips as well as that on the palm (see Figure 10B) become stable and the final grasp is achieved.

Regarding the hand trajectory, it is interesting to notice that the joint value of the first phalanges of Fingers 2 and 4 (*q*4 and *q*10) stays almost constant for a few milliseconds around *t* = 0.15 s, that is when the contact with the first phalanges of Fingers 2 and 4 is detected.

Figure 11A shows the translation of the object in the world reference frame, which can be seen in Figure 5B. The *x*-axis is along the direction of closure of the Fingers 2 and 4, the *y*-axis is in the direction connecting the bases of the Fingers 2 and 4, and the *z*-axis is normal to the plane of the hand palm and points upwards. As expected, the sphere is dragged in the positive *x* direction. It initially tends to go downwards due to the contact force exerted by the first phalanges of Fingers 2 and 4, but then comes back upwards transported by the fingertips. The motion in the *y* direction is null, whereas the rotation with respect to it is the only change of orientation that is observed for the sphere (see the *θ* angle in Figure 11B).

### 4.2 Objects Made of Different Materials

This set of simulations demonstrates that even though the gravity is not activated, changing the inertial properties of the object influences its resulting motion when it is grasped by the gripper. In particular, the higher the density, the less the object tends to go downward in the *z* direction, and the closer it arrives to Fingers 2 and 4 at the end of the simulation (Figure 12A,C). Fore more dense materials, not only the translation, but also the rotation is more limited (Figure 12B).

## 5 Conclusions and Future Work

In this paper, we introduced a numerical tool for the simulation of robotic grasping using Simulink and Simscape Multibody. The tool is based on SynGrasp, a previously developed MATLAB toolbox allowing purely kinematics or quasi-static analyses. The library of functions and blocks developed in this work adds a fundamental module enabling dynamics simulations. The Simscape Multibody model of a robotic hand can be defined in a straightforward way from SynGrasp models thanks to a series of functions based on programmatic model editing. In this way, even users with limited experience with the Simscape Multibody environment can build robotic hand models and run dynamics simulations. More expert users can improve hand models, for example importing realistic CAD representations of links and structural components, or introducing specific mechanical transmission systems, or completing the model with advanced hand control systems. Grasped objects can be selected among a series of primitive geometric shapes, or can be defined by means of their 3D CAD representation. Contact detection and contact force evaluation are defined by means of a specific block.

The developed library is presented and its features are demonstrated through a set of simulations performed using a multifingered gripper interacting with different objects. In the presented simulations, to better highlight the basic system characteristics, the effect of gravity was not considered. In particular, contact point detection was analysed, since it represents the most challenging part of the simulation, in terms of numerical complexity and in terms of result stability and reliability. In the first set of simulations, grasps with different objects were simulated, whereas in the second set we investigated grasping of objects with the same shapes but different inertial properties. The obtained results were coherent with the expected system behaviour in all the simulated cases.

Future developments of this work are manifold. To make the simulation more realistic, we will introduce the gravity, and the contact detection between the links of the hand themselves. In addition, we will study how to simulate the simultaneous grasping of multiple objects, and we will introduce the environment in the simulations. In this way, grasping strategies exploiting environmental constraints (Bimbo et al., 2019; Pozzi et al., 2020) could be tested. A deeper investigation on contact modeling and detection will also be conducted. As it is implemented now, the contact detection only allows retrieving the phalanx in which the contact was detected, and not the exact contact point on the phalanx surface. For this, it would be necessary to explicitly build and use a contact mesh. In addition, other contact models will be considered and possibly compared, depending on the type of simulated interactions and tasks (Skrinjar et al., 2018).

We will also consider the inclusion of blocks evaluating, during the simulation, specific grasp properties (e.g., grasp robustness, manipulability indices, grasp quality measures, etc.). Since the toolbox is capable of performing numerical dynamic simulations, it is particularly suitable to model and simulate soft robotic hands (Pozzi et al., 2018). The dynamic equations governing the motion of soft bodies are highly nonlinear and in most cases cannot be solved analytically in a closed form. For this reason, one must resort to the numerical solution of the resulting dynamic equations. We also plan to implement the complete simulation of the hand-arm system in grasping and manipulation tasks, possibly introducing dual-arm systems capable of executing complex bimanual tasks.

## Data Availability

The original contributions presented in the study are included in the article/Supplementary Material, further inquiries can be directed to the corresponding author.

## Footnotes

1
https://www.mscsoftware.com/it/product/adams

2
https://www.ode.org/

3
https://opensim.stanford.edu/

4
https://www.mbdyn.org/?Software_Download

5
http://www.freedyn.at/new-download/

6
https://www.ansys.com/it-it/products/structures/ansys-motion

7
https://www.comsol.com/multibody-dynamics-module

8
https://www.solidworks.com/

9 Programmatic Model Editing (MathWorks, Inc., Natick, Massachusetts, United States): https://it.mathworks.com/help/simulink/programmatic-modeling.html

10 Notice that since human and robotic hands are usually modelled with only revolute joints, also in this work we consider only this type of joints. In principle, however, the implementation could be easily extended to include also prismatic joints, as they are available within Simscape Multibody.

11 To maintain the connection with the hand models defined in SynGrasp, we assume that phalanges have cylindrical shapes. However, the models can be customized to account for different designs.

12
https://it.mathworks.com/help/physmod/sm/ref/spatialcontactforce.html.

13 Notice that although we chose realistic values for the density, we did not aim at replicating the properties of specific types of materials, as this would require the adjustment also of other material properties and this is out of the scope of this work.

## References

[B1] Achilli G. M. Logozzo S. Valigi M. C. Malvezzi M. (2021a). “Preliminary Study on Multibody Modeling and Simulation of an Underactuated Gripper with Differential Transmission,” in International Design Engineering Technical Conferences and Computers and Information in Engineering Conference (New York, NY, USA: American Society of Mechanical Engineers (ASME)), 85468, V009T09A005. 10.1115/detc2021-72162 10.1115/detc2021-72162 | Google Scholar

[B2] Achilli G. M. Logozzo S. Valigi M. C. Salvietti G. Prattichizzo D. Malvezzi M. (2021b). “Underactuated Soft Gripper for Helping Humans in Harmful Works,” in International Workshop IFToMM for Sustainable Development Goals (Cham: Springer), 264–272. 10.1007/978-3-030-87383-7_29 10.1007/978-3-030-87383-7_29 | Google Scholar

[B3] Achilli G. M. Valigi M. C. Salvietti G. Malvezzi M. (2020). Design of Soft Grippers with Modular Actuated Embedded Constraints. Robotics 9, 105. 10.3390/robotics9040105 10.3390/robotics9040105 | Google Scholar

[B4] Ahn D.-S. (2014). Integrated Solidworks & Simscape Platform for the Model-Based Control Algorithms of Robot Manipulators. J. Korea Soc. Power Syst. Eng. 18, 91–96. 10.9726/kspse.2014.18.4.091 10.9726/kspse.2014.18.4.091 | Google Scholar

[B5] Aldair A. A. Al-Mayyahi A. Jasim B. H. (2020). “Control of Eight-Leg Walking Robot Using Fuzzy Technique Based on Simscape Multibody Toolbox,” in IOP Conference Series: Materials Science and Engineering (IOP Publishing), 745, 012015. 10.1088/1757-899x/745/1/012015 10.1088/1757-899x/745/1/012015 | Google Scholar

[B6] Allard J. Cotin S. Faure F. Bensoussan P.-J. Poyer F. Duriez C. (2007). “Sofa-an Open Source Framework for Medical Simulation,” in MMVR 15-Medicine Meets Virtual Reality (IOP Press), 125, 13–18. Google Scholar 17377224

[B7] Almeida L. Moreno P. (2020). “Potential Grasp Robustness for Underactuated Hands: New Heuristics and Uncertainty Considerations,” in 2020 IEEE International Conference on Autonomous Robot Systems and Competitions (ICARSC), Ponta Delgada, Portugal (IEEE), 233–238. 10.1109/icarsc49921.2020.9096152 10.1109/icarsc49921.2020.9096152 | Google Scholar

[B8] Almeida L. Moreno P. (2021). Uncertainty and Heuristics for Underactuated Hands: Grasp Pose Selection Based on the Ppotential Grasp Robustness Metric. SN Appl. Sci. 3, 1–15. 10.1007/s42452-021-04594-5 10.1007/s42452-021-04594-5 | Google Scholar

[B9] Bimbo J. Turco E. Ghazaei Ardakani M. Pozzi M. Salvietti G. Bo V. (2019). Exploiting Robot Hand Compliance and Environmental Constraints for Edge Grasps. Front. Robot. AI 6, 135. 10.3389/frobt.2019.00135 PubMed Abstract | 10.3389/frobt.2019.00135 | Google Scholar 33501150 PMC7805883

[B10] Calli B. Walsman A. Singh A. Srinivasa S. Abbeel P. Dollar A. M. (2015). Benchmarking in Manipulation Research: Using the Yale-Cmu-Berkeley Object and Model Set. IEEE Robot. Autom. Mag. 22, 36–52. 10.1109/MRA.2015.2448951 10.1109/MRA.2015.2448951 | Google Scholar

[B11] Collins J. Chand S. Vanderkop A. Howard D. (2021). A Review of Physics Simulators for Robotic Applications. IEEE Access 9, 51416–51431. 10.1109/access.2021.3068769 10.1109/access.2021.3068769 | Google Scholar

[B12] Corke P. Haviland J. (2021). “Not your Grandmother’s Toolbox–The Robotics Toolbox Reinvented for python,” in 2021 IEEE International Conference on Robotics and Automation (ICRA), Xi’an, China (IEEE), 11357–11363. Google Scholar

[B13] Corke P. (2017). Robotics, Vision and Control: Fundamental Algorithms in MATLAB® Second, Completely Revised, 118. Cham: Springer. Google Scholar

[B14] Corral E. Gismeros Moreno R. G. Meneses J. García M. J. G. Castejón C. (2021). Spatial Algorithms for Geometric Contact Detection in Multibody System Dynamics. Mathematics 9, 1359. 10.3390/math9121359 10.3390/math9121359 | Google Scholar

[B15] De Simone M. C. Russo S. Rivera Z. B. Guida D. (2017). “Multibody Model of a Uav in Presence of Wind Fields,” in 2017 International Conference on Control, Artificial Intelligence, Robotics & Optimization (ICCAIRO) (IEEE), 83–88. 10.1109/iccairo.2017.26 10.1109/iccairo.2017.26 | Google Scholar

[B16] Eldirdiry O. Zaier R. (2018). “Modeling Biomechanical Legs with Toe-Joint Using Simscape,” in 2018 11th International Symposium on Mechatronics and its Applications (ISMA), Sharjah, United Arab Emirates (IEEE), 1–7. 10.1109/isma.2018.8330129 10.1109/isma.2018.8330129 | Google Scholar

[B17] Fattahi Sani M. Abeywardena S. Psomopoulou E. Ascione R. Dogramadzi S. (2019). “Towards Finger Motion Tracking and Analyses for Cardiac Surgery,” in Mediterranean Conference on Medical and Biological Engineering and Computing (Cham: Springer), 1515–1525. 10.1007/978-3-030-31635-8_188 10.1007/978-3-030-31635-8_188 | Google Scholar

[B18] Garate V. R. Pozzi M. Prattichizzo D. Tsagarakis N. Ajoudani A. (2018). Grasp Stiffness Control in Robotic Hands through Coordinated Optimization of Pose and Joint Stiffness. IEEE Robot. Autom. Lett. 3, 3952–3959. 10.1109/lra.2018.2858271 10.1109/lra.2018.2858271 | Google Scholar

[B19] Garate V. R. Tsagarakis N. Bicchi A. Ajoudani A. (2017). “On the Common-Mode and Configuration-dependent Stiffness Control of Multiple Degrees of Freedom Hands,” in 2017 IEEE-RAS 17th International Conference on Humanoid Robotics (Humanoids) (IEEE), 113–120. 10.1109/humanoids.2017.8239545 10.1109/humanoids.2017.8239545 | Google Scholar

[B20] Gioioso G. Salvietti G. Malvezzi M. Prattichizzo D. (2013). Mapping Synergies from Human to Robotic Hands with Dissimilar Kinematics: an Approach in the Object Domain. IEEE Trans. Robot. 29, 825–837. 10.1109/tro.2013.2252251 10.1109/tro.2013.2252251 | Google Scholar

[B21] Hosea M. E. Shampine L. F. (1994). Efficiency Comparisons of Methods for Integrating Odes. Comput. Math. Appl. 28, 45–55. 10.1016/0898-1221(94)00151-0 10.1016/0898-1221(94)00151-0 | Google Scholar

[B22] Katyara S. Ficuciello F. Caldwell D. G. Siciliano B. Chen F. (2021). Leveraging Kernelized Synergies on Shared Subspace for Precision Grasping and Dexterous Manipulation. IEEE Trans. Cogn. Dev. Syst. 1. 10.1109/TCDS.2021.3110406 10.1109/TCDS.2021.3110406 | Google Scholar

[B23] Khnissi K. Jabeur C. B. Seddik H. (2019). “3d Simulator for Navigation of a Mobile Robot Using Simscape-Simulink,” in 2019 International Conference on Control, Automation and Diagnosis (ICCAD), Grenoble, France (IEEE), 1–6. 10.1109/iccad46983.2019.9037958 10.1109/iccad46983.2019.9037958 | Google Scholar

[B24] Koenig N. Howard A. (2004). “Design and Use Paradigms for Gazebo, an Open-Source Multi-Robot Simulator,” in IEEE/RSJ International Conference on Intelligent Robots and Systems, Sendai, Japan. (IEEE), 2149–2154. Google Scholar

[B25] Le Ngoc T. Nguyen T. L. (2020). Quasi-physical Modeling of Robot Irb 120 Using Simscape Multibody for Dynamic and Control Simulation. Turkish J. Electr. Eng. Comput. Sci. 28, 1949–1964. Google Scholar

[B26] Logozzo S. Valigi M. C. (2019). “Investigation of Instabilities in Mechanical Face Seals: Prediction of Critical Speed Values,” in IFToMM World Congress on Mechanism and Machine Science (Cham: Springer), 3865–3872. 10.1007/978-3-030-20131-9_383 10.1007/978-3-030-20131-9_383 | Google Scholar

[B27] Machado M. Moreira P. Flores P. Lankarani H. M. (2012). Compliant Contact Force Models in Multibody Dynamics: Evolution of the Hertz Contact Theory. Mech. Mach. Theory 53, 99–121. 10.1016/j.mechmachtheory.2012.02.010 10.1016/j.mechmachtheory.2012.02.010 | Google Scholar

[B28] Malvezzi M. Gioioso G. Salvietti G. Prattichizzo D. (2015). Syngrasp: A Matlab Toolbox for Underactuated and Compliant Hands. IEEE Robot. Autom. Mag. 22, 52–68. 10.1109/mra.2015.2408772 10.1109/mra.2015.2408772 | Google Scholar

[B29] Malvezzi M. Iqbal Z. Valigi M. C. Pozzi M. Prattichizzo D. Salvietti G. (2019). Design of Multiple Wearable Robotic Extra Fingers for Human Hand Augmentation. Robotics 8, 102. 10.3390/robotics8040102 10.3390/robotics8040102 | Google Scholar

[B30] Malvezzi M. Valigi M. C. Salvietti G. Iqbal Z. Hussain I. Prattichizzo D. (2018). “Design Criteria for Wearable Robotic Extra-fingers with Underactuated Modular Structure,” in The International Conference of IFToMM ITALY (Cham: Springer), 509–517. 10.1007/978-3-030-03320-0_56 10.1007/978-3-030-03320-0_56 | Google Scholar

[B31] Marullo S. Pozzi M. Malvezzi M. Prattichizzo D. (2022). Analysis of Postures for Handwriting on Touch Screens without Using Tools. Sci. Rep. 12, 296. 10.1038/s41598-021-04367-5 PubMed Abstract | 10.1038/s41598-021-04367-5 | Google Scholar 34997155 PMC8741930

[B32] Meattini R. Chiaravalli D. Palli G. Melchiorri C. (2021). Exploiting In-Hand Knowledge in Hybrid Joint-Cartesian Mapping for Anthropomorphic Robotic Hands. IEEE Robot. Autom. Lett. 6, 5517–5524. 10.1109/lra.2021.3078658 10.1109/lra.2021.3078658 | Google Scholar

[B33] Miller A. T. Allen P. K. (2004). Graspit!: A Versatile Simulator for Grasp Analysis. IEEE Robotics & Automation Magazine. Google Scholar

[B34] Noskievič P. Walica D. (2020). “Design and Realisation of the Simulation Model of the Stewart Platform Using the Matlab-Simulink and the Simscape Multibody Library,” in 2020 21th International Carpathian Control Conference (ICCC), High Tatras, Slovakia (IEEE), 1–5. 10.1109/iccc49264.2020.9257249 10.1109/iccc49264.2020.9257249 | Google Scholar

[B35] Olaya J. Pintor N. Avilés O. F. Chaparro J. (2017). Analysis of 3 Rps Robotic Platform Motion in Simscape and Matlab Gui Environment. Int. J. Appl. Eng. Res. 12, 1460–1468. Google Scholar

[B36] Orabona A. Palazzi A. Graziosi S. Ferrise F. Bordegoni M. (2020). “Design of a Simplified 3d-Printed Artificial Underactuated Hand,” in Proceedings of the Design Society: DESIGN Conference (Cambridge, England: Cambridge University Press), 1, 1027–1036. 10.1017/dsd.2020.311 10.1017/dsd.2020.311 | Google Scholar

[B37] Pozzi M. Malvezzi M. Prattichizzo D. (2017). On Grasp Quality Measures: Grasp Robustness and Contact Force Distribution in Underactuated and Compliant Robotic Hands. IEEE Robotics Automation Lett. 2, 329–336. 10.1109/lra.2016.2612304 10.1109/lra.2016.2612304 | Google Scholar

[B38] Pozzi M. Marullo S. Salvietti G. Bimbo J. Malvezzi M. Prattichizzo D. (2020). Hand Closure Model for Planning Top Grasps with Soft Robotic Hands. Int. J. Robotics Res. 39, 1706–1723. 10.1177/0278364920947469 10.1177/0278364920947469 | Google Scholar

[B39] Pozzi M. Miguel E. Deimel R. Malvezzi M. Bickel B. Brock O. (2018). “Efficient Fem-Based Simulation of Soft Robots Modeled as Kinematic Chains,” in Proceedings IEEE International Conference on Robotics and Automation, ICRA, Brisbane, QLD, Australia. (IEEE), 1–8. 10.1109/icra.2018.8461106 10.1109/icra.2018.8461106 | Google Scholar

[B40] Prattichizzo D. Pozzi M. Malvezzi M. (2020). “Dexterous Manipulation,” in Encyclopedia of Robotics. Editors Ang H. M. Khatib O. Siciliano B. (Berlin, Heidelberg: Springer). 10.1007/978-3-642-41610-1_180-1 10.1007/978-3-642-41610-1_180-1 | Google Scholar

[B41] Qian W. Xia Z. Xiong J. Gan Y. Guo Y. Weng S. (2014). “Manipulation Task Simulation Using Ros and Gazebo,” in 2014 IEEE International Conference on Robotics and Biomimetics (ROBIO 2014), Bali, Indonesia (IEEE), 2594–2598. 10.1109/robio.2014.7090732 10.1109/robio.2014.7090732 | Google Scholar

[B42] Ruiz Garate V. Ajoudani A. (2020). An Approach to Object-Level Stiffness Regulation of Hand-Arm Systems Subject to Under-actuation Constraints. Aut. Robots 44, 1505–1517. 10.1007/s10514-020-09942-9 10.1007/s10514-020-09942-9 | Google Scholar

[B43] Shabana A. A. (2003). Dynamics of Multibody Systems. Cambridge, England: Cambridge University Press. Google Scholar

[B44] Shigley J. E. (1972). Mechanical Engineering Design. McGraw-Hill Companies. Google Scholar

[B45] Siciliano B. Sciavicco L. Villani L. Oriolo G. (2009). Robotics: Modeling, Planning and Control. Springer. Google Scholar

[B46] Siwek M. Baranowski L. Panasiuk J. Kaczmarek W. (2019). “Modeling and Simulation of Movement of Dispersed Group of Mobile Robots Using Simscape Multibody Software,” in AIP Conference Proceedings (AIP Publishing LLC), 2078, 020045. 10.1063/1.5092048 10.1063/1.5092048 | Google Scholar

[B47] Skrinjar L. Slavič J. Boltežar M. (2018). A Review of Continuous Contact-Force Models in Multibody Dynamics. Int. J. Mech. Sci. 145, 171–187. 10.1016/j.ijmecsci.2018.07.010 10.1016/j.ijmecsci.2018.07.010 | Google Scholar

[B48] Taghizadegan A. Piltan F. Sulaiman N. B. (2016). Design High Frequency Surgical Robot Controller: Design Fpga-Based Controller for Surgical Robot Manipulator Simscape Modeling. Ijhit 9, 431–474. 10.14257/ijhit.2016.9.5.37 10.14257/ijhit.2016.9.5.37 | Google Scholar

[B49] Tselegkaridis S. Sapounidis T. (2021). Simulators in Educational Robotics: A Review. Educ. Sci. 11, 11. 10.3390/educsci11010011 10.3390/educsci11010011 | Google Scholar

[B50] Udai A. D. Rajeevlochana C. Saha S. K. (2011). “Dynamic Simulation of a Kuka Kr5 Industrial Robot Using Matlab Simmechanics,” in 15th National Conference on Machines and Mechanisms, Chennai, India, 96, 1–8. Google Scholar

[B51] Ulbrich S. Kappler D. Asfour T. Vahrenkamp N. Bierbaum A. Przybylski M. (2011). “The Opengrasp Benchmarking Suite: An Environment for the Comparative Analysis of Grasping and Dexterous Manipulation,” in 2011 IEEE/RSJ International Conference on Intelligent Robots and Systems, San Francisco, CA, USA (IEEE), 1761–1767. 10.1109/iros.2011.6094894 10.1109/iros.2011.6094894 | Google Scholar

[B52] Valigi M. C. Logozzo S. Malvezzi M. (2020a). “Design and Analysis of a Top Locking Snap Hook for Landing Manoeuvres,” in The International Conference of IFToMM ITALY (Cham: Springer), 484–491. 10.1007/978-3-030-55807-9_55 10.1007/978-3-030-55807-9_55 | Google Scholar

[B53] Valigi M. C. Logozzo S. Meli E. Rindi A. (2020b). New Instrumented Trolleys and a Procedure for Automatic 3d Optical Inspection of Railways. Sensors 20, 2927. 10.3390/s20102927 PubMed Abstract | 10.3390/s20102927 | Google Scholar PMC7285245 32455726

[B54] Yao K. Billard A. (2020). An Inverse Optimization Approach to Understand Human Acquisition of Kinematic Coordination in Bimanual Fine Manipulation Tasks. Biol. Cybern. 114, 63–82. 10.1007/s00422-019-00814-9 PubMed Abstract | 10.1007/s00422-019-00814-9 | Google Scholar 31907609 PMC7062861

[B55] Zaidi L. Corrales J. A. Bouzgarrou B. C. Mezouar Y. Sabourin L. (2017). Model-based Strategy for grasping3Ddeformable Objects Using a Multi-Fingered Robotic Hand. Robotics Aut. Syst. 95, 196–206. 10.1016/j.robot.2017.06.011 10.1016/j.robot.2017.06.011 | Google Scholar

[B56] Zhu Y. Liu Y. Zhang L. Wang Y. Niu W. Huang C. (2022). Dynamic Model and Motion Characteristics of an Underwater Glider with Manta-Inspired Wings. J. Bionic Eng. 19, 1–15. 10.1007/s42235-021-00130-8 10.1007/s42235-021-00130-8 | Google Scholar

